# Association between sleep quality, migraine and migraine burden

**DOI:** 10.3389/fneur.2022.955298

**Published:** 2022-08-26

**Authors:** Shaojie Duan, Zhiying Ren, Hui Xia, Ziyao Wang, Tao Zheng, Zunjing Liu

**Affiliations:** ^1^Graduate School of Beijing University of Chinese Medicine, Beijing, China; ^2^Department of Neurology, China-Japan Friendship Hospital, Beijing, China; ^3^Department of Neurology, Peking University People's Hospital, Beijing, China

**Keywords:** sleep quality, migraine, migraine burden, Pittsburgh Sleep Quality Index, association

## Abstract

**Background:**

The relationship between sleep and migraine is well known to be bidirectional. However, few studies have systematically assessed the association between sleep quality and the risk of developing migraine, and its gender and age differences are unclear. And there is currently limited evidence on the associations between sleep quality and migraine-related burdens.

**Objective:**

The objectives of this study were to: (1) explore the association between sleep quality and the risk of developing migraine, and its gender and age differences; (2) investigate the associations between sleep quality and the total pain burden, severity, disability, headache impact, quality of life, anxiety, and depression of migraine patients.

**Methods:**

This study consecutively enrolled 134 migraine patients and 70 sex- and age-matched healthy control subjects. Sleep quality was assessed through the Pittsburgh Sleep Quality Index (PSQI). Logistic regression and linear regression analyses were used to explore the associations between sleep quality with the risk of developing migraine and the migraine-related burdens.

**Results:**

The prevalence of poor sleep quality in migraine patients was significantly higher than that in subjects without migraine (*P* < 0.001). After adjusting for various confounding factors, the risk of migraine with poor sleep quality remained 3.981 times that of those with good sleep quality. The subgroup analysis showed that there were significant additive interactions between poor sleep quality and the risk of migraine in gender, age, and education level (*P* for interaction < 0.05), and the stronger correlations were found in females, populations with ages more than 35 years old, and with lower education levels. In addition, multivariate linear regression analysis showed that poor sleep quality was significantly and independently associated with the total pain burden, severity, headache impact, quality of life, anxiety, and depression in migraine patients (*P* trend < 0.05).

**Conclusion:**

Poor sleep quality was significantly independently associated with an increased risk of developing migraine and the migraine-related burdens. Strengthening PSQI assessment is valuable for the early prevention and treatment of migraine patients.

## Introduction

Migraine is one of the most common chronic neurological disorders characterized by attacks of moderate or severe headaches and reversible neurological and systemic symptoms (1, 2). WHO ranks migraine as the third most prevalent medical condition and the second most disabling neurological disorder in the world (3, 4). The annual and lifetime prevalence was 18% and 33% in women and 6% and 13% in men, respectively (1). The prevalence peaks between the ages of 35 and 39 years (5). Migraine can reduce the health-related quality of life and considerable disability, and their condition has a substantial effect on daily activities, direct medical costs, and prevalence of medical comorbidities (1, 6, 7), which lead to a substantial burden on themselves and the entire family (8, 9). Despite the availability of evidence-based guidelines intended to inform clinical decision-making with migraine, the management of migraine for the population remains suboptimal (1, 10). Therefore, early detection of factors strongly associated with migraine and migraine-related burdens can identify the potential interventions and approaches, with important implications for migraine prevention, treatment, and prognosis.

Migraine is well known to be associated with a wide range of sleep disturbances (11). Recently, the bidirectional relationship between sleep and migraine has especially received more and more research and attention (12–14). Poor sleep quality, as one of the most common sleep disturbances, is more prevalent in patients with migraine than in the general population and has an important impact on migraine chronicity (15, 16). In addition, it has been suggested that poor sleep quality may precede migraine attacks (17). However, the association between sleep quality and the risk of developing migraine is not fully understood. Considering the gender and age differences of migraine, whether the association between sleep quality and the risk of migraine is also affected by age and gender requires further research. In addition, there is currently limited evidence on the associations between sleep quality and the total pain burden, severity, disability, headache impact, quality of life, and anxiety and depression in migraine patients.

Pittsburgh Sleep Quality Index (PSQI) is a common self-reported sleep quality questionnaire used to assess sleep quality over the past month and has been used in many studies to assess poor sleep quality in migraine patients (18). Considering the close relationship between sleep quality and migraine, the PSQI score may be a potential predictor of migraine. However, few studies have reported the predictive value of the PSQI score for migraine.

Therefore, this study first aimed to systematically explore the relationship between sleep quality and the risk of developing migraine, as well as its gender and age differences, and then to investigate the associations between sleep quality and the total pain burden, severity, disability, headache impact, quality of life, and anxiety and depression in migraine patients. In addition, we also initially assessed the predictive value of the PSQI score on migraine, aiming to provide new ideas for early prevention and screening of migraine.

## Materials and methods

### Study design and participants

The design of this study mainly consists of two parts. First, we conducted a case-control study to investigate the association between sleep quality and the risk of developing migraine, as well as the gender and age differences in the association. According to the diagnosis criteria of migraine by International Classification of Headache Disorders, 3rd edition (ICHD-III) (19), 134 migraine subjects (including 11 with aura and 123 without aura) were consecutively enrolled from the headache clinic in the Department of Neurology at China-Japan Friendship Hospital between February 2021 and March 2022. At the same time, we also recruited 70 sex- and age-matched healthy volunteers without a previous diagnosis of other primary or secondary headache disorders during the same period.

Then, we conducted a further cross-sectional analysis of these 134 migraine patients to investigate the associations between sleep quality and the total pain burden, severity, disability, headache impact, quality of life, and anxiety and depression in migraine patients. All subjects received the professional diagnostic assessment and completed the standardized questionnaire interviewed by a certified neurologist and headache specialist. All subjects accepted to participate in this study voluntarily and signed informed consent.

### Data collection

Through the interview with standardized questionnaires, we collected all subjects' basic materials such as age, gender, height, weight, smoking history, drinking history, exercise time, subjective pressure, and education level and assessed their sleep quality and emotional disorder, including anxiety and depression. As for the migraine patients, we further assessed their total pain burden, severity, disability, and quality of life. Body mass index (BMI) was calculated as the weight (in kilograms) divided by the square of the height (in meters). The definition of smoking history and drinking history referred to the current or previous behavior of smoking and drinking. Weekly exercise time referred to the total exercise time (hour) per week. Subjective pressure referred to the inner stress that subjects feel about their studies, work, or life, as assessed by a visual analog scale pressure score on a scale of 0 to 10. Education level was divided into undergraduate and below, and graduate and above.

### Pittsburgh sleep quality index

The Pittsburgh Sleep Quality Index (PSQI) is a common self-rated questionnaire to assess sleep quality (20). It included 19 items in seven components, including subjective sleep quality, sleep latency, sleep time, habitual sleep efficiency, sleep disorders, sleep medication, and daytime dysfunction (21). Poor sleep quality is defined when PSQI score is >5, with a diagnostic sensitivity of 98.7 and specificity of 84.4 (22).

### Zung's self-rating anxiety scale and self-rating depression scale

Zung's Self-rating Anxiety Scale (SAS) and Self-rating Depression Scale (SDS) are norm-referenced scales to assess anxiety and depression, respectively (23), and their reliability and validity have been validated in the Chinese population (24, 25). Both the scales include 20 items, with raw scale scores ranging from 20 to 80 and the transformed index score ranging from 25 to 100, and a higher score indicates a higher level of mental disorder. According to previous studies using SAS and SDS scores to assess anxiety and depression in the Chinese population, a total transformed score of 53 or 50 was defined as a cut-off value of depression or anxiety, respectively (26, 27).

### Visual analog scale pain score

The visual analog scale (VAS) pain score is one of the most widely used to assess the subjective pain intensity of patients with migraine (28, 29). The pain intensity was determined by asking, “On a scale of 0 to 10 (where 0 = no pain at all and 10 = pain as bad as it can be), on average how painful are your headaches?”

### Total pain burden

Total pain burden of migraine is a composite measure involving frequency, duration, and severity, calculated by multiplying the duration (hours) of migraine headache, and maximum pain severity (0 = none, 1= mild, 2 = moderate, 3 = severe) for each migraine headache day and summing these over the days in a month, indicating the monthly severity-weighted duration (30).

### Migraine disability assessment questionnaire

The Migraine Disability Assessment (MIDAS) is a 5-item questionnaire used to quantify migraine-related disability (31), which has been proven in previous studies to have high reliability and high internal consistency (32, 33). A score of 0 to 5 indicates little or no disability, a score of 6 to 10 indicates mild disability, a score of 11 to 20 indicates moderate disability, and a score of ?21 indicates severe disability (34).

### Headache impact test

The Headache Impact Test (HIT-6) is a self-report questionnaire designed to assess the impact of headaches on quality of life (35), and its validity and reliability have been validated in patients with various types of headaches, including primary and secondary headaches (36), episodic and chronic migraine (37–39). The questionnaire consists of six items where cumulative total score ranges from 36 to 78 points, higher scores indicate a greater impact of headaches on the patient's life, and it can be divided into four grades according to this (40).

### Migraine-specific quality-of-life questionnaire

The Migraine-Specific Quality-of-Life Scale (MSQ) (version 2.1) is one of the most widely used measures of the impact of migraine on quality of life that consists of three components, including role function-restrictive, role function-preventive, and emotional function (41). A higher cumulative total score indicates a better quality of life for migraine patients. In this study, we used the Migraine-Specific Quality-of-Life Questionnaire Chinese version 2.1 (MSQv2.1-C), which exhibited satisfactory reliability and validity in a sample of individuals with migraine who speak Chinese (42).

### Statistical analysis

First, the baseline characteristics of the migraine and control groups were compared. The independent-samples *t*-test was used for comparing normally distributed quantitative data between groups. The Mann–Whitney *U*-test was used for comparing non-normally distributed quantitative data between groups. The chi-squared test was used for comparing categorical data between groups. Then, multivariate logistic regression analysis was conducted to evaluate the effect of sleep quality on the risk of migraine under different adjustment conditions and different subgroups. The receiver operating characteristic (ROC) curve was used to evaluate the predictive ability of the PSQI score for migraine in different subgroups, and the DeLong test was conducted to compare the predictive ability of the PSQI score and other related indicators for migraine. In addition, multivariate linear regression analysis was further performed for migraine patients to estimate the associations between sleep quality and the total pain burden, VAS score, MIDAS score, HIT-6 score, MSQ score, SAS score, and SDS score.

All statistical tests were two-tailed and were considered significant for *P* < 0.05 (*P* < 0.05). Statistical analyses were performed using Statistical Package for the Sciences (SPSS, version 25.0) and MedCalc statistical software (version 19.6.4).

## Results

### Baseline characteristics of participants

There were 115 females and 19 males in the migraine group, with an average age of 36.63 ± 8.84 years, and there were 60 females and 10 males in the control group, with an average age of 34.67 ± 8.97 years. There was no statistically significant difference in gender and age between the two groups, and they were comparable. The baseline characteristics of the two groups of subjects are presented in Table 1. The results showed that the educational level of migraine patients was significantly lower than that of the control group (*P* < 0.001). However, the PSQI score, SAS score, SDS score levels, and the prevalence of poor sleep quality, anxiety, and depression were significantly higher than those of the control group (all *P* < 0.05).

**Table 1:** Baseline characteristics of migraine and control groups.

**Variable**	**Total (*N* = 204)**	**Control (*N* = 70)**	**Migraine (*N* = 134)**	*P-*value^*^
Gender [*n* (%)]				0.983
Male	29 (14.2%)	10 (14.3%)	19 (14.2%)	
Female	175 (85.8%)	60 (85.7%)	115 (85.8%)	
Age, years (mean±SD)	35.96 ± 8.91	34.67 ± 8.97	36.63 ± 8.84	0.059
BMI, kg/m^2^ (mean±SD)	21.78 ± 3.83	21.66 ± 4.54	21.84 ± 3.40	0.774
Smoking history [n (%)]	16 (7.8%)	3 (4.3%)	13 (9.8%)	0.272
Drinking history [n (%)]	41 (20.1%)	15 (21.4%)	26 (19.5%)	0.751
Weekly exercise time, hours (mean±SD)	0.86 ± 1.84	0.93 ± 2.09	0.83 ± 1.70	0.782
Pressure score (mean±SD)	5.25 ± 2.39	5.37 ± 2.34	5.19 ± 2.42	0.638
Education level [*n* (%)]				<0.001
Undergraduate and below	100 (49.0%)	6 (8.6%)	94 (70.1%)	
Graduate and above	104 (51.0%)	64 (91.4%)	40 (29.9%)	
PSQI score [M (P25, P75)]	6.0 (4.0,8.0)	4.0 (3.0,5.3)	7.0 (5.0,9.0)	<0.001
Poor sleep quality [*n* (%)]	106 (52.0%)	17 (24.3%)	89 (66.9%)	<0.001
SAS score [M (P25, P75)]	38.8 (32.5,46.3)	35.0 (30.0,39.1)	42.5 (35.0,48.8)	<0.001
Anxiety [*n* (%)]	31 (15.2%)	4 (5.7%)	27 (20.1%)	0.007
SDS score [M (P25, P75)]	38.8 (32.5,50.0)	36.3 (31.3,43.8)	40.0 (33.8,51.3)	0.012
Depression [*n* (%)]	39 (19.1%)	7 (10%)	32 (23.9%)	0.017

BMI, body mass index; PSQI, Pittsburgh Sleep Quality Index; SAS, Self-rating Anxiety Scale; SDS, Self-rating Depression Scale.

* Categorical variables were calculated by chi-square test, and Fisher's exact test was used when the sample size was <5. Quantitative variables were calculated by the Mann–Whitney U-test or independent-samples t-test.

### Effect of sleep quality on the risk of developing migraine

Multivariate logistic regression analyses were conducted to explore the effect of sleep quality on the risk of developing migraine, and the results are presented in Table 2. PSQI score had a strong association with migraine, and the odds ratio (OR) for a 1-SD increase in PSQI score was 1.657 (95%CI: 1.397–1.965) without adjustment (Model 1). After adjusting for age, gender, smoking history, drinking history, BMI, weekly exercise time, pressure score, education level, SAS score, and SDS score, there was still a 1.626-fold (95%CI: 1.312–2.015) higher risk for migraine with a 1-SD increase in PSQI score (Model 4).

**Table 2:** Multivariate logistic regression analysis of sleep quality on the risk of migraine.

	**Variable**	**β**	**SE**	**Wald *χ^2^***	***P-*value**	**OR (95%CI)**
Model 1	PSQI score	0.505	0.087	33.586	<0.001	1.657(1.397, 1.965)
	Poor sleep quality	1.842	0.334	30.372	<0.001	6.306(3.276, 12.139)
Model 2	PSQI score	0.503	0.087	33.249	<0.001	1.654(1.394, 1.963)
	Poor sleep quality	1.830	0.334	29.967	<0.001	6.235(3.238, 12.007)
Model 3	PSQI score	0.469	0.087	29.342	<0.001	1.599(1.349, 1.895)
	Poor sleep quality	1.687	0.339	24.806	<0.001	5.404(2.782, 10.496)
Model 4	PSQI score	0.486	0.109	19.741	<0.001	1.626(1.312, 2.015)
	Poor sleep quality	1.381	0.431	10.285	0.001	3.981(1.711, 9.260)

PSQI, Pittsburgh sleep quality index. Model 1: unadjusted. Model 2: adjusted for age and gender. Model 3: adjusted for age, gender, smoking history, drinking history, BMI, weekly exercise time, and pressure score. Model 4: adjusted for age, gender, smoking history, drinking history, BMI, weekly exercise time, pressure score, education level, SAS score, and SDS score.

After dividing all participants into good sleep quality subgroup and poor sleep quality subgroup according to PSQI score, the risk of migraine increased robustly in the poor sleep quality subgroup, with the OR value of 6.306 (95%CI: 3.276–12.139) to 3.981 (95%CI: 1.711–9.260) from Model 1 to Model 4.

### Effect of poor sleep quality on the risk of developing migraine stratified by subgroups

To further investigate the impact of other risk factors on the correlation between poor sleep quality and migraine risk, subgroup analyses were carried out according to gender, age, smoking history, drinking history, anxiety, depression, and education level. Table 3 summarizes the results of the subgroup analysis and the interaction results. After adjusting for age, gender, smoking history, drinking history, BMI, weekly exercise time, pressure score, education level, SAS score, and SDS score, there were still significant additive interactions between poor sleep quality and migraine risk in gender, age, and education level (*P* for interaction <0.05). Stronger correlations were found in participants with an age > 35 years old, lower education level, and females. However, significant interactions were not found in the smoking history, drinking history, anxiety, and depression subgroups.

**Table 3:** Effect of poor sleep quality on migraine risk stratified by subgroups.

**Subgroups**	**No. of participation**	**OR(95%CI)**	***P-*value**	***P* for interaction**
Age				0.001
>35 y	100	11.478 (2.674, 49.275)	0.001	
≤ 35 y	104	2.556 (0.615, 10.627)	0.197	
Gender				0.002
Males	29	2.302 (0.054, 98.012)	0.663	
Females	175	4.461 (1.726, 11.527)	0.002	
Smoking history				0.580
Yes	16	-	-	
No	188	4.368 (1.761, 10.835)	0.001	
Drinking history				0.239
Yes	41	43.282 (0.236, 7,934.661)	0.156	
No	163	3.583 (1.357, 9.456)	0.010	
Anxiety				0.392
Yes	31	0.387(0,-)	1	
No	173	4.323 (1.682, 11.105)	0.002	
Depression				0.219
Yes	39	0(0,-)	0.991	
No	165	4.496 (1.677, 12.057)	0.003	
Education level				0.018
Undergraduate and below	100	229.912 (1.061, 49,827.01)	0.048	
Graduate and above	104	3.216 (1.258, 8.225)	0.015	

Adjusted for age, gender, smoking history, drinking history, BMI, weekly exercise time, pressure score, education level, SAS score, and SDS score.

### Predictive ability of PSQI score for migraine

**Figure 1:**
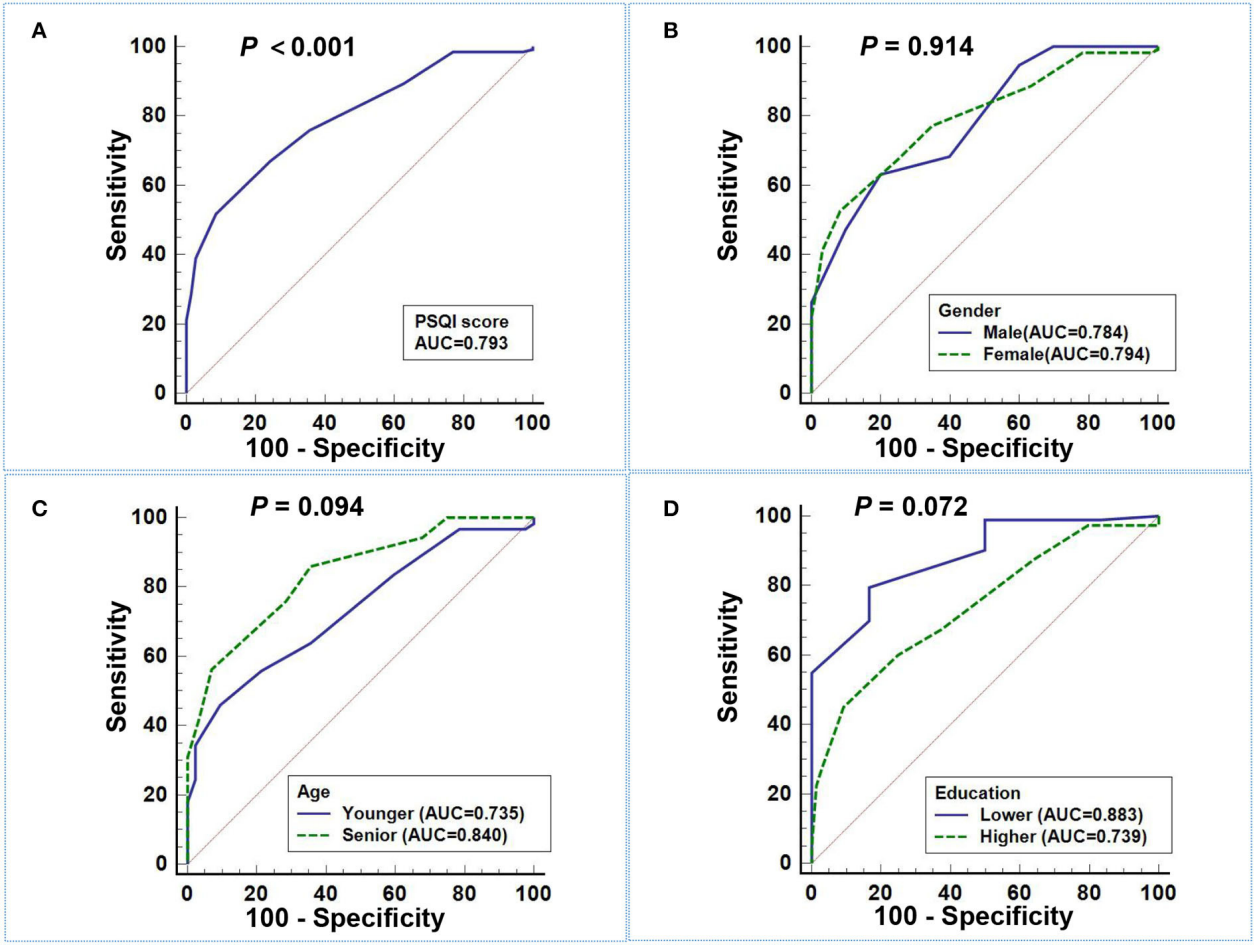
ROC curve of PSQI score in predicting migraine. **(A)** The AUC of PSQI score in predicting migraine in total subjects was 0.793 (95%CI: 0.731–0.847). **(B–D)** There were no significant differences in the AUC of PSQI score to predict migraine among different gender **(B)**, age **(C)**, and education **(D)** subgroups (all *P* > 0.05).

The ROC curve of PSQI score in predicting migraine in different gender, ages, education subgroups, and total subjects is plotted in Figure 1, and the DeLong test was used to compare the area under the ROC curve (AUC) between the subgroups. The PSQI score had a good predictive ability for migraine, with an AUC of 0.793 (95%CI: 0.731–0.847), and the sensitivity and specificity were 51.88% and 91.43%, separately. However, subgroup comparison found that there were no significant differences in the AUC of PSQI score to predict migraine in the subgroups (all *P* > 0.05).

In addition, we also compared the predictive ability for migraine among PSQI score and its seven components, as well as SAS and SDS scores. Table 4 illustrates the ROC curve results, and the AUC of PSQI score to predict migraine was significantly higher than its seven components, including subjective sleep quality, sleep latency, sleep time, habitual sleep efficiency, sleep disorders, sleep medication, and daytime dysfunction (all *P* < 0.01). Moreover, the predictive ability of the PSQI score for migraine was significantly higher than that of the SDS score, but there was no statistical difference compared with the SAS score.

**Table 4:** ROC curve results of PSQI score and related indicators in predicting migraine.

**Variable**	**AUC (95%CI)**	**Sensitivity (%)**	**Specificity (%)**	***P*-value**
PSQI score	0.793 (0.731–0.847)	51.88	91.43	<0.0001
Subjective sleep quality	0.667 (0.598–0.731)^*^	47.76	80.00	<0.0001
Sleep latency	0.655 (0.585–0.720)^*^	39.85	84.29	<0.0001
Sleep time	0.538 (0.467–0.608)^*^	30.60	87.14	0.2620
Habitual sleep efficiency	0.640 (0.570–0.706)^*^	39.85	88.57	<0.0001
Sleep disorders	0.674 (0.605–0.738)^*^	93.28	36.23	<0.0001
Sleep medication	0.512 (0.441–0.582)^*^	8.21	94.20	0.5319
Daytime dysfunction	0.555 (0.484–0.624)^*^	20.15	91.43	0.1617
SAS score	0.725 (0.660–0.786)	61.94	75.71	<0.0001
SDS score	0.608 (0.537–0.675)^*^	41.79	77.14	0.0093

PSQI, Pittsburgh Sleep Quality Index; SAS, Self-rating Anxiety Scale; SDS, Self-rating Depression Scale. Compared with PSQI score,

* Significantly lower than the AUC of the PSQI score.

### Associations between sleep quality and migraine-related burdens

**Figure 2:**
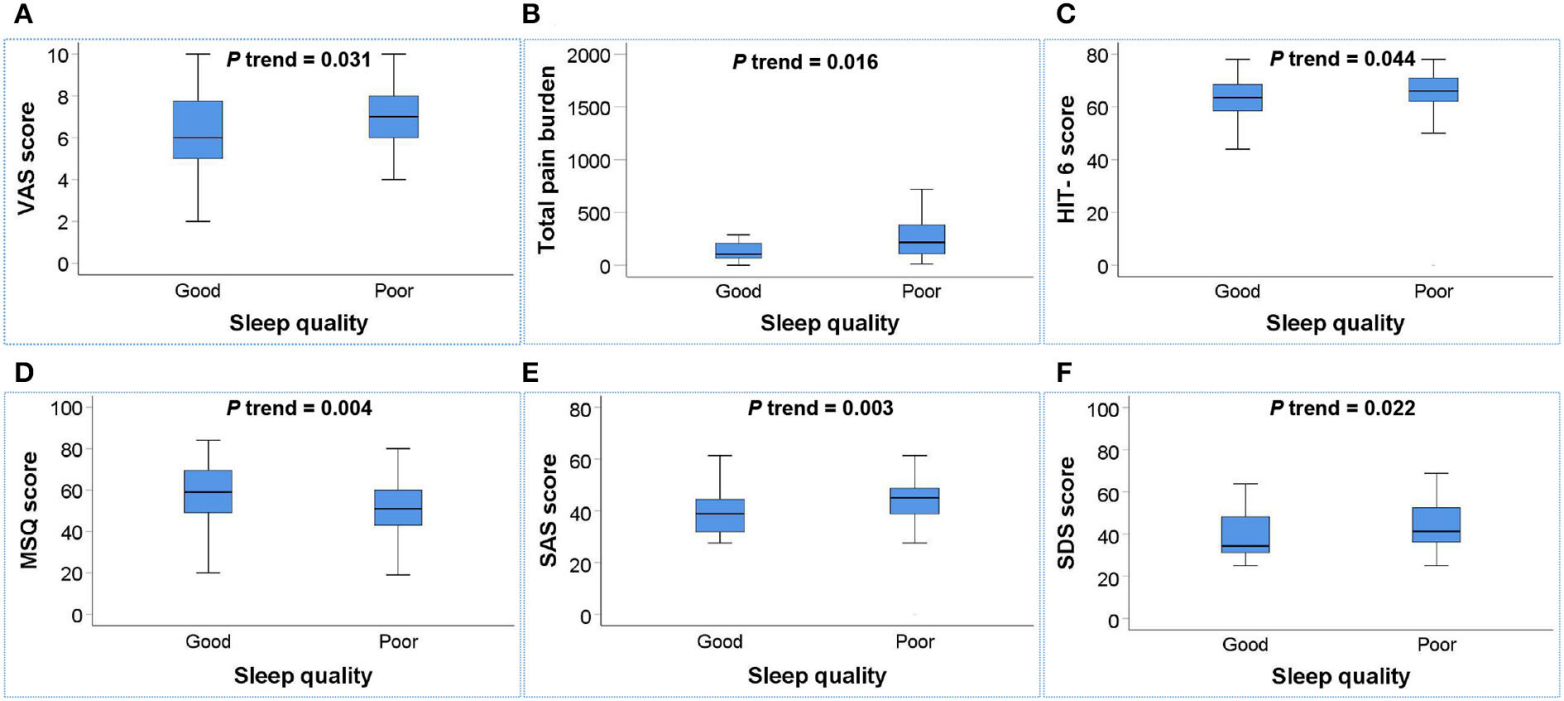
Box plot of the relationship between sleep quality and migraine-related burdens. **(A–F)** Poor sleep quality was positively associated with the total pain burden **(B)**, VAS score **(A)**, HIT-6 score **(C)**, SAS score **(E)**, and SDS score **(F)** and negatively associated with MSQ score **(D)** in migraine patients (all *P* trend < 0.05). *P* trend value: Adjusted for age, gender, smoking history, drinking history, BMI, weekly exercise time, pressure score, education level, anxiety, and depression.

To further investigate the associations between sleep quality and migraine-related burdens, we conducted multivariate linear regression analysis for 134 migraine patients to explore the associations between sleep quality and the total pain burden, VAS score, HIT-6 score, MIDAS score, MSQ score, SAS score, and SDS score, and the results are illustrated in Table 5 and Figure 2. After adjusting for age, gender, smoking history, drinking history, BMI, weekly exercise time, pressure score, education level, anxiety, and depression, the poor sleep quality was still significantly associated with the total pain burden, VAS score, HIT-6 score, MSQ score, SAS score, and SDS score, indicating that poor sleep quality was independently associated with the total pain burden, severity, headache impact, quality of life, and anxiety and depression in migraine patients.

**Table 5:** Multivariate linear regression analysis of the effects of poor sleep quality on migraine-related indicators.

**Variable**	**Good sleep quality (*N* = 45)**	**Poor sleep quality (*N* = 89)**	*P* trend^*^
VAS score	6.489 ± 1.844	7.202 ± 1.563	0.031
HIT-6 score	62.750 ± 9.640	65.310 ± 9.505	0.044
MIDAS score	25.545 ± 31.141	31.352 ± 33.032	0.865
MSQ score	58.160 ± 14.999	49.750 ± 14.984	0.004
SAS score	38.436 ± 10.205	43.5393 ± 9.541	0.003
SDS score	37.955 ± 11.595	44.199 ± 12.120	0.022
Total pain burden	183.296 ± 256.295	314.236 ± 311.614	0.016

VAS, visual analog scale; HIT-6, Headache Impact Test; MIDAS, Migraine Disability Assessment; MSQ, Migraine-Specific Quality-of-Life Questionnaire; SAS, Self-rating Anxiety Scale; SDS, Self-rating Depression Scale.

* Adjusted for age, gender, smoking history, drinking history, BMI, weekly exercise time, pressure score, education level, anxiety, and depression.

## Discussion

This study systematically analyzed the associations between sleep quality with the risk of developing migraine and the migraine-related burdens, aiming to provide new clinical evidence for the prevention and treatment of migraine. The result showed that about two-thirds of migraine patients had poor sleep quality, and the poor sleep quality was significantly associated with an increased risk of migraine, especially in females, populations with ages more than 35 years old, and lower education levels. At the same time, the poor sleep quality was independently associated with the total pain burden, severity, headache impact, quality of life, anxiety, and depression in migraine patients. In addition, this study initially found that the PSQI score had good diagnostic specificity for migraine and may be used as a reference index to predict migraine, which provided a new idea for early prevention and screening of migraine.

A growing number of scholars have focused on the impact of sleep on migraine, and preliminary studies have been conducted on the relationship between them (12, 13, 17, 43). Studies from several population-based studies in Korea have shown that people with migraine or probable migraine have significantly higher insomnia (44), poor sleep quality (45), and insufficient sleep duration (46) compared to non-migraine populations. A meta-analysis also reported that migraine patients have significantly poorer subjective sleep quality and altered sleep architecture compared to healthy individuals (18). Similar findings were obtained in this study, and there was a significantly higher level of PSQI score and prevalence of poor sleep quality among subjects in the migraine group. Multivariate logistic regression showed that poor sleep quality remained an independent risk factor for migraine after adjusted for various confounding factors, and the risk of developing migraine in poor sleep quality was still 3.981 times higher than that in good sleep quality, which further confirmed the strong positive association between poor sleep quality and increased migraine risk.

It has been suggested that there is a bidirectional relationship between migraine and sleep (47), and the bidirectional relationship between sleep and migraine may occur on several levels. First, the intersection between trigeminal pain-signaling networks and neural networks regulating arousal and sleep in multiple levels of the cerebral cortex, thalamus, hypothalamus, and brainstem is the anatomical and physiological basis for sleep and migraine (43, 47). The pathophysiology of migraine includes the activation and sensitization of the trigeminal vascular system, cortical spreading depression (CSD), and excitation–inhibition imbalances in the dura, brainstem, cortical, and subcortical regions (2, 48, 49). However, the firing of cortical neurons is influenced by the duration of wakefulness or sleep, with reduced firing rates and synchrony after sustained sleep (50, 51). In addition, sleep deprivation can lead to an increased CSD susceptibility that promotes the occurrence of migraine (43). Second, changes in the concentration of certain neurotransmitters and biochemical factors are common regulatory pathways and mediators of sleep and migraine. Especially, 5-hydroxytryptaminergic neurons play a variety of important roles in sleep control including promoting wakefulness, initiating and maintaining sleep (52, 53), and abnormal cerebral vasodilation and contraction, altered vascular permeability, and disruption of the pain modulation system due to dysregulated 5-hydroxytryptamine levels are among the pathophysiological mechanisms of migraine (48, 54, 55). Moreover, the concentration of the vasodilator adenosine increases with the duration of sleep deprivation and during migraine attacks, and the precipitating effect of adenosine administration on migraine attacks has been reported (49, 56, 57). In addition, the orexin system and melatonin system are also closely and intrinsically linked to sleep and migraine (47).

Compared with other previous studies, this study also took into account the gender, age, and other differences in migraine and further conducted a subgroup analysis to explore whether the association between sleep quality and migraine risk was affected by gender, age, or other factors. The results showed that the effect of sleep quality on the risk of migraine was more pronounced in participants older than 35 years and in women. Regarding gender differences, migraines are about three times more common in women than in men, and the reasons for this are mainly related to hormone levels (58, 59). Fluctuations in estrogen levels affect several serotonins, including 5-hydroxytryptamine (60), which in turn affects migraine and sleep quality, and may be one of the reasons for the gender differences in migraine risk affected by sleep quality. As they age, migraine patients show age-related specific metabolic changes in the brain (61), and the sleep EEG functional connectivity varies that is also strongly affected by the age (62), which may be one of the reasons explaining the age-related impact between sleep quality and migraine risk. Interestingly, this study also found that low education level was one of the factors that affect the association between poor sleep quality and the risk of migraine, which will provide new ideas for migraine prevention and treatment for people with different educational levels. In addition, it would be interesting to assess whether the association between sleep quality and migraine is different according to whether patients are in treatment with migraine preventive treatments, but the small sample size of healthy control subjects in this study using preventive therapy does not allow for subgroup analysis. Therefore, we hope to further expand the sample size in the future to explore the effect of preventive treatment on the association between sleep quality and migraine.

The associations between sleep quality with migraine-related burdens are also important topics worth focusing on (7, 11, 12, 17). Previous studies have shown that poor sleep quality can increase the headache-related effects and indirectly increase the frequency and severity of migraine (63). The results of the Migraine in America Symptoms and Treatment (MAST) study also showed that the headache pain intensity and frequency of migraine are closely associated with insomnia (64). However, previous studies that considered migraine burden tended to focus only on migraine frequency or severity, which may have underestimated the total migraine burden by ignoring the duration of pain. Therefore, this study used the total pain burden that included the frequency, severity, and duration, as well as VAS score, MIDAS score, HIT-6 score, MSQ score, SAS score, and SDS score to provide a more comprehensive assessment of migraine-related burdens. The results showed that poor sleep quality was independently associated with the total pain burden, VAS score, HIT-6 score, MSQ score, SAS score, and SDS score, indicating that sleep quality played a crucial role in the total pain burden, severity, headache impact, quality of life, anxiety, and depression in migraine patients. Although multivariate linear regression analysis did not show a significant independent linear trend between poor sleep quality and MIDAS score, migraine patients with poor sleep quality still had a higher level of MIDAS score. These results confirmed the significant associations between poor sleep quality and migraine-related burdens, which will provide new ideas for the prevention and treatment of migraine-related burdens.

In addition, considering the significant association of PSQI score with the risk of developing migraine and that PSQI was convenient, practical, and accurate in assessing sleep quality, this study also initially explored the predictive value of the PSQI score for migraine. The result showed that the PSQI score had good predictive accuracy and diagnostic specificity for migraine, and it may be a potential predictor for the occurrence of migraine. However, this study was not a prospective study and cannot validate its predictive power, and further validation is needed in the future.

In summary, this study confirmed the significant associations between poor sleep quality with an increased risk of developing migraine and the migraine-related burdens and initially found the potential predictive value of the PSQI score for migraine, which will provide a piece of new clinical evidence for early prevention and sleep intervention of migraine. However, there were still some limitations in our study. First, subjects in this study did not undergo polysomnography, but used PSQI to assess sleep quality, which may be relatively subjective. Second, the sample size of male subjects in this study was relatively small; although it reached the minimum sample size required for statistics, the positive result of gender difference may be amplified due to selection bias. Third, this study used a cross-sectional approach to investigate the relationship between sleep quality and migraine-related burdens in 134 migraine patients, which was unable to demonstrate a causal relationship between them. In addition, the relatively small number of migraine patients with aura prevented further subgroup analyses to explore the association differences between sleep quality and migraine-related burdens in migraine patients with and without aura. Therefore, a multicenter, larger sample prospective cohort study is required in the future for further comprehensive assessment of causal relationships and subtype differences between sleep quality with migraine and its burden.

## Conclusions

Poor sleep quality was significantly independently associated with an increased risk of developing migraine and the migraine-related burdens. Strengthening PSQI assessment is valuable for the early prevention and treatment of migraine patients.

## Data availability statement

The raw data supporting the conclusions of this article will be made available by the authors, without undue reservation.

## Ethics statement

Ethical review and approval was not required for the study on human participants in accordance with the local legislation and institutional requirements. Written informed consent for participation was not required for this study in accordance with the national legislation and the institutional requirements.

## Author contributions

SD: study concept and design and drafting of the manuscript. SD, ZR, HX, ZW, and TZ: acquisition of data. SD, ZR, and HX: analysis and interpretation of data. ZL: revising manuscript for intellectual content. SD and ZL: final approval of the completed manuscript. All authors contributed to the article and approved the submitted version.

## Funding

This study was supported by the Elite Medical Professionals project of China-Japan Friendship Hospital (NO.ZRJY 2021-BJ03).

## Conflict of interest

The authors declare that the research was conducted in the absence of any commercial or financial relationships that could be construed as a potential conflict of interest.

## Publisher's note

All claims expressed in this article are solely those of the authors and do not necessarily represent those of their affiliated organizations, or those of the publisher, the editors and the reviewers. Any product that may be evaluated in this article, or claim that may be made by its manufacturer, is not guaranteed or endorsed by the publisher.

## Abbreviations

PSQI: Pittsburgh Sleep Quality Index
ROC: receiver operating characteristic
AUC: area under the ROC curve
ICHD-III: International Classification of Headache Disorders, 3rd edition
BMI: body mass index
SAS: Self-rating Anxiety Scale
SDS: Self-rating Depression Scale
VAS: visual analog scale
HIT-6: Headache Impact Test
MIDAS: Migraine Disability Assessment
MSQ: Migraine-Specific Quality-of-Life Questionnaire.
